# Enhancing Fetal Cardiac Care: Addressing Unwarranted Variation in Prenatal Congenital Heart Disease Detection and Counseling

**DOI:** 10.1007/s11886-026-02369-1

**Published:** 2026-04-18

**Authors:** Bethan A. Lemley, Mariem Abdelsalam, Jennifer Co-Vu, Pei-Ni Jone, Cameron Kalin, Aparna Kulkarni, Joyce L. Woo

**Affiliations:** 1 Division of Cardiology, The Ann and Robert H. Lurie Children’s Hospital of Chicago, Chicago, IL USA; 2 Feinberg School of Medicine, Northwestern University, Chicago, IL USA; 3 UF Health Congenital Heart Center, University of Florida College of Medicine, Gainesville, FL USA; 4 Northwell Health, Cohen Children’s Medical Center, Cohen Children’s Heart Center, New Hyde Park, NY USA

**Keywords:** Access to care, Artificial intelligence, Congenital heart disease, Prenatal diagnosis, Prenatal counseling, Fetal echocardiogram

## Abstract

**Purpose of Review:**

To summarize sources of variation in prenatal diagnosis of congenital heart disease (CHD) in the United States and low- and middle-income countries, and to review current and emerging interventions aimed at reducing this variation.

**Recent Findings:**

In the United States, approximately half of infants requiring cardiac surgery before 6 months of age receive a prenatal diagnosis of congenital heart disease. There is substantial variation in this rate domestically and globally. This variation reflects two primary contributors: socioeconomic disparities and clinical practice variation. Strategies to reduce unwarranted variation include standardized ultrasonography guidelines, local policy interventions, and emerging technologies including artificial intelligence and tele-echocardiography.

**Summary:**

Successful prenatal cardiac diagnosis encompasses three domains: (1) accessing fetal cardiac care, (2) obtaining accurate anatomic assessment, and (3) and receiving comprehensive counseling. Addressing socioeconomic disparities and clinical practice variation could reduce overall variation and increase prenatal diagnosis rates.

## Introduction

Congenital heart defects (CHDs) are the most common type of birth defects worldwide and incur the most resources [1–3]. In the United States, fetal echocardiography at experienced centers approaches 90% sensitivity [4–7], but prenatal CHD diagnosis rates are lower. This discrepancy reflects significant variation in prenatal diagnosis rates across subpopulations: a national cross-sectional analysis using the Society of Thoracic Surgeons Congenital Heart Surgery Database, including patients who had congenital heart surgery before 6 months of age from 2006 to 2023, reported an overall prenatal diagnosis rate of approximately 48%. However, this varied widely by geographic region (43.6–56.2%), and lesion type (13.1–77.1%) [7].

Reducing both variation and disparities is important for improving overall prenatal diagnosis rates and equitably achieving the best outcomes. Early prenatal diagnosis plays a key role for familial outcomes by enabling decision-making regarding pregnancy management as well as delivery timing and location [8, 9]. It facilitates comprehensive, multidisciplinary counseling with pediatric cardiology, maternal–fetal medicine, neonatology, genetics, palliative care and other subspecialties. For many families, multidisciplinary counseling enhances understanding of the spectrum of medical and surgical treatment options, clarifies prognosis, and supports fully informed decision-making regarding pregnancy continuation versus termination (depending on local laws and cultural norms), or pursuit of a hospice birth plan (e.g., comfort care or palliative care) [10].

Prenatal diagnosis also influences postnatal outcomes by enabling risk-appropriate neonatal care, including delivery planning and initiation of prostaglandin E1 for ductal-dependent lesions [11]. Prenatal diagnosis is associated with lower preoperative morbidity and mortality [12, 13] and facilitates timely, life-saving interventions [14, 15] which in turn is associated with better postoperative and neurodevelopmental outcomes [16, 17]. It also has significant implications for postnatal costs by decreasing the number of emergency neonatal transfers to tertiary care centers for diagnosis and intervention, reducing both financial costs and the psychosocial burden of separating neonates from their parents [18].

**Fig. 1:**
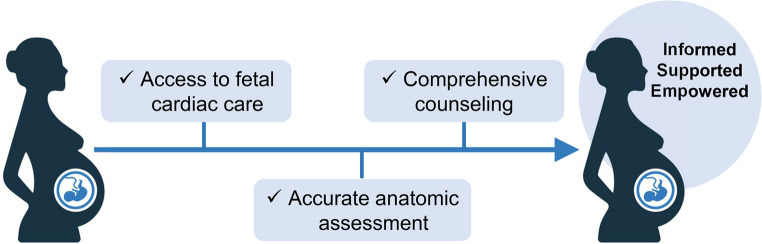
Conceptual framework for prenatal diagnosis of congenital heart disease. The key steps in successful prenatal diagnosis of congenital heart disease include access to fetal cardiac care, accurate anatomic assessment, and comprehensive counseling. (iStock.com/Bethan A. Lemley/Joyce L. Woo.)

Given the profound impact of prenatal diagnosis on familial and postnatal outcomes, it is essential to address the factors underlying the significant variation in prenatal diagnosis. This variation arises from two sources of sub-variation: differences in clinical practice, and disparities, a socially-defined subset of variation that may be inequitable or unjust [19]. Thus, henceforth we use the term *disparity* for socially defined differences in prenatal diagnosis rates, and *variation* for differences attributable to clinical practice. We discuss disparities and variation, within and outside the United States, using a conceptual framework of three domains that collectively enable effective prenatal diagnosis: (1) access to fetal cardiac care, (2) accurate anatomic assessment, and (3) comprehensive counseling (Fig. 1). Within each domain, we also summarize existing and emerging interventions to reduce unwarranted variation in prenatal diagnosis rates.

## Access to Fetal Cardiac Care

### Disparities in Prenatal Diagnosis in the United States

National disparities in prenatal diagnosis rates include the birthing patient’s insurance type, ethnicity, distance to care, and neighborhood-level socioeconomic context. In a national retrospective cohort study of fetuses and infants who underwent surgery for transposition of the great arteries or hypoplastic left heart syndrome from 2012 to 2016, Krishnan et al. reported that lower neighborhood socioeconomic status, Hispanic ethnicity, and rural residence were all associated with decreased likelihood of prenatal diagnosis [20]. Another study using nationwide Medicaid claims from 2007 to 2012 also demonstrated an association between neighborhood socioeconomic status and prenatal diagnosis [21].

Smaller regional studies, which often leverage more granular data, revealed more specifics about local disparities in prenatal diagnosis. Davtyan et al. reported that in southern California, prenatal CHD diagnosis rate was 58% among infants who received cardiac intervention prior to 6 months old from 2016 to 2019. Having health insurance and identifying as Hispanic ethnicity were associated with receiving a prenatal diagnosis, but insurance type (public versus private), neighborhood socioeconomic status, and distance from a fetal cardiologist were not [22]. In contrast, a study from Boston reported a prenatal diagnosis rate of 50% among infants who underwent surgery before 30 days of age from 2003 to 2006, and found that insurance type and neighborhood socioeconomic status were strongly associated with prenatal diagnosis, while race was not [23]. A study from Oregon evaluated factors associated with gestational age at first fetal echo from 2017 to 2018, and found no associations with ethnicity, zip code, rurality, or distance to hospital [24].

Further, interactions between socioeconomic factors introduce further complexity in prenatal diagnosis disparities. Woo et al. investigated prenatal diagnosis among neonates who underwent surgery for critical congenital heart disease from 2020 to 2021, in Illinois. For those patients who lived within Chicago city limits, there was no association between insurance type and prenatal CHD diagnosis, but for those who lived outside of the city, public insurance was associated with a lower probability of prenatal diagnosis than private insurance [25]. To further delineate causes of insurance-based disparities in prenatal diagnosis, the authors also found that receipt of second-trimester anatomy ultrasound explained 39% of the association between insurance type and prenatal diagnosis, whereas receipt of fetal echocardiogram did not explain any of the insurance-based disparity [26]. These studies emphasize the importance of regional data to inform interventions and policy, and the importance of granular data to delineate the complex mechanisms that drive socioeconomic disparities.

### Addressing Disparities in Prenatal Diagnosis in the United States

Addressing disparities in neighborhood socioeconomic status, insurance status, and distance to care may seem daunting for the individual clinician. However, such disparities are driven, in part, by modifiable barriers. In a single-center, mixed-methods study on patient-reported barriers to prenatal CHD diagnoses, limited appointment availability, absence of appointment reminders, multiple transfers of care, lack of transportation, long distance to fetal echocardiogram, difficulty with insurance approval, and challenges in securing childcare for other children were all identified as significant barriers [27]. These barriers represent more feasible targets for clinicians, hospitals, and local policy makers to address. Examples of actionable interventions that have already shown promise in other areas of healthcare include subsidized transportation and lodging for patients who commute long distances for care [28], text and email-based appointment reminders [29, 30], and statewide mandates for short-term disability to attend appointments without forgone income [31].

Telemedicine and tele-echocardiography represent promising interventions to address several barriers to prenatal diagnosis [32, 33]. Some studies suggest that when tele-echocardiography is performed with proper equipment, trained sonographers, and skilled remote interpreters (MFM physicians, fetal cardiologists), it can approach the diagnostic performance of in-person interpretation [34, 35]. A single-center, randomized trial comparing remote tele-interpreted obstetric ultrasound to standard in-person ultrasound demonstrated non-inferiority of tele-interpretation [36]. In addition to mitigating distance-related barriers in access to prenatal diagnosis, tele-echocardiography has also been shown to be highly cost-effective among “standard-risk” pregnant individuals [37]. In the post-COVID era, telemedicine and tele-echocardiography is an appealing tool to increase access to fetal cardiac care [38].

### Disparities in Prenatal Diagnosis Outside the United States

Globally, there is substantial variation in prenatal CHD detection rates between countries. High-income countries generally report higher rates of prenatal diagnosis than low-income countries. After a prenatal screening program was introduced in 2007, the prenatal CHD diagnosis rate in the Netherlands rose to 60% [39]. Waern et al. reported a rate of 56% in Sweden among infants requiring intervention [40]. In contrast, prenatal diagnosis rates in sub-Saharan Africa, Afghanistan, and Yemen are estimated around 20% [41].

Studies from low- and middle-income countries, including Egypt, Nepal, Indonesia, and several sub-Saharan African nations describe barriers to prenatal diagnosis that are distinct from those reported in high-income countries, including limited financial and infrastructural resources, shortages of trained expertise such as the lack of pediatric cardiologists, insufficient opportunities for sonographer training, and lack of awareness of the utility of fetal echocardiography [42, 43].

Awareness of the utility of fetal echocardiography is a key, rate-limiting barrier that may be unique to low- and middle-income countries. A survey of parents visiting an outpatient pediatric cardiology clinic of a tertiary care center in India, who may be more knowledgeable about CHD care than those who do not reach a tertiary center, showed that only 2.2% of respondents had heard of fetal echocardiography [44]. In another survey study from Egypt of 200 parents of children admitted to a pediatric cardiology unit, 67% were unaware of any prenatal diagnostic options for CHD [45].

## Accurate Anatomic Assessment

### Variation Due to Clinician and Sonographer Characteristics

Variation in prenatal diagnosis can be introduced by the heterogeneity of clinician specialty [46–48]. Across the United States, maternal-fetal medicine specialists (MFM) often perform fetal echocardiograms, especially for high-risk pregnancies or after referral from an obstetrician (OB). However, in some geographic areas, radiologists with expertise in fetal ultrasound may perform fetal echocardiograms in collaboration with MFM or OB services [49]. At many tertiary or academic centers, pediatric cardiologists, often with advanced training in imaging, perform fetal echocardiography. Institutional resources, availability and distribution of subspecialists determine which subspecialists perform fetal cardiac screening [48, 50]. To mitigate potential specialty-based variation, the American Institute of Ultrasound in Medicine (AIUM) published training guidelines for the performance of fetal echocardiography in 2020, followed by an update by the American Society of Echocardiography (ASE) in conjunction with the American College of Cardiology and the American Heart Association in 2023 [49, 51]. All guidelines recommended that ‘‘only well trained or experienced pediatric cardiologists, maternal-fetal medicine specialists, obstetricians, or radiologists who have acquired the appropriate knowledge base and skills should supervise and perform fetal echocardiograms’’ and that ongoing quality improvement efforts be documented [49]. Familiarity with indications, limitations, principles of ultrasound, anatomy, physiology and pathophysiology along with appropriate documentation methods have been recommended as standards for clinical practice of fetal echocardiography [51, 52].

When an abnormality is suspected, referral to a fetal cardiologist may be necessary to obtain a more extensive study with comprehensive counseling regarding postnatal management and outcomes. In some centers, an ultrasound sonographer with appropriate training performs the scan, but images are interpreted by fetal cardiologists. To mitigate this additional potential source of variation in prenatal detection, the AIUM endorses that fetal cardiac sonographers should be appropriately credentialed in accordance with AIUM accreditation policies. Furthermore, physicians must provide supervision, as defined by the Centers for Medicare and Medicaid Services Code of Federal Regulations.

### Variation Due to Timing of and Indication for Fetal Cardiology Referral

Accurate anatomic assessment also depends on timing of fetal echocardiography. Fetal echocardiography is generally recommended at 18 to 22 weeks of gestation to achieve optimal image quality. However, advances in ultrasound technology now permit fetal cardiac evaluation as early as 12 to 14 weeks gestation [49]. Early diagnosis (before 15 weeks) offers several advantages including additional time for decision-making about pregnancy management, further evaluation for associated anomalies, and an opportunity to gain insight into CHD evolution from earlier in gestation [49]. Nevertheless, early fetal echocardiography may be limited by lower sensitivity and greater variability in image quality, therefore, a low threshold for repeat echocardiography at 18 to 22 weeks gestation may be warranted to confirm and refine the diagnosis.

Indications for fetal echocardiogram include those populations with a CHD risk ≥ 2 to 3%, including those with an abnormal obstetric ultrasound (e.g., suspected cardiac abnormality or fetal arrhythmia), high-risk condition (e.g., pre-gestational diabetes or teratogenic exposure), or family history (e.g., first degree relative with CHD, or suspected chromosomal abnormality) [46, 51]. The degree of adherence to established indications for fetal echocardiography may also introduce variation in reported prenatal detection rates. A retrospective analysis at a tertiary center in Karachi, Pakistan, found that a large proportion of fetal echocardiography referrals were based on indications not strictly supported by international guidelines; potentially contributing to a relatively low detection rate [53].

### Variation Due to Equipment and Patient Characteristics

Additional considerations for variation in fetal echocardiography quality include the ultrasound systems, which require high spatial and temporal resolution given the small size of fetal cardiac structures and rapid heart rate. For transabdominal scanning, curvilinear probes are optimal given their wide near-field view and near parallel ultrasound beams. The transducer frequency range should be 2 to 7 MHz for late second- and third-trimester scans, while 5 to 12 MHz may be useful for late first-trimester and early second-trimester scans. Two-dimensional (2D; B-mode), M-mode, color flow and pulsed wave Doppler ultrasound are minimum technological requirements. Continuous wave Doppler ultrasound may characterize high flow velocities. Adjustments in frequency, harmonics, sector width, and depth can optimize frame rate and lateral resolution at the necessary depth [49].

A significant degree of variation in prenatal diagnosis is due to CHD lesion type, ranging from 13.1 to 77.1% [7]. To mitigate this variation, society guidelines recommend that all fetal echocardiograms include a sequential segmental analysis that includes the situs, atria, ventricles, and great arteries. These standardized outflow views assess the great arteries, specifically ventriculo-arterial connections, vessel size, patency, velocity and direction, size of aortic isthmus and ductus arteriosus, pulmonary artery bifurcation, and spatial relationships among the transverse aortic arch, ductus arteriosus, and trachea [49, 51]. The addition of standardized outflow views has substantially decreased variation in prenatal diagnosis and increased overall diagnosis rates by 40 to 50% [54, 55].

Patient-related characteristics such as increased maternal body habitus, obesity, multiparity, plurality, and abdominal scar tissue can lead to suboptimal image quality due to the attenuation of ultrasound waves, representing another source of variation in fetal echocardiographic quality. Adding to this challenge, higher plurality, maternal obesity and diabetes are also associated with higher risk of abnormal fetal cardiac structure and function [56, 57].

### Future Directions to Reduce Variation in Anatomic Assessment

Artificial intelligence (AI) has the potential to mitigate some of the above sources of variation in fetal echocardiogram quality and interpretation by distinguishing normal versus abnormal findings. Arnaout et al. used deep learning, specifically a convolutional neural network, to detect complex fetal CHD from normal cardiac anatomy, achieving 95% sensitivity and 96% specificity [58]. Zelop et al. demonstrated how AI-based software could identify severe CHD on second-trimester ultrasound with a high sensitivity of 0.97 [59]. Meta-analyses report a pooled 0.89 sensitivity and 0.9 specificity for binary classification (normal vs. abnormal) with area under the curve (AUC) up to 0.99 in large datasets [60]. These algorithms may be particularly useful in minimizing diagnostic variation for clinicians who are less experienced in CHD screening, including those practicing outside tertiary centers or those serving vulnerable populations in resource-limited settings [61]. For those practicing in tertiary centers that specialize in CHD care, assistance from AI software may also reduce time in obtaining standard views and allow additional time for the sonographer to focus on abnormal cardiac pathology [62]. AI may also reduce interpretation time, thus potentially increasing time for counseling or increasing the number of appointments available at tertiary centers [63]. Nurmaini et al. used deep learning to improve the effectiveness of routine prenatal screening for major CHD. They used the DenseNet 201 model to achieve 99% sensitivity, 97% specificity, and 98% accuracy for inter-patient scenario, a robust way to validate AI models [64]. Further refinement to generalize AI models is needed in multicenter, prospective studies prior to real-world implementation.

## Comprehensive Counseling

### Variation and Disparities in Communication

Comprehensive counseling is a key component of prenatal diagnosis, where variability may be introduced in effectively communicating the diagnosis and prognosis of CHD. Several studies have demonstrated variation in knowledge gaps between the information that counseling clinicians give and what parents perceive [65–68], which may be partly exacerbated by education and language-based disparities. Holmes et al. found that maternal education was positively correlated with accuracy of maternal description of the fetal cardiac diagnosis [66]. Kovacevic et al. conducted a multi-center survey study in Germany and found that parental language (native language other than German) and age influenced trust in medical staff and perceived situational control [68]. The authors commented that language barriers may directly impact parents’ understanding of the cardiac anatomy but could also exacerbate cultural differences affecting the patient’s trust in the counseling clinician. A qualitative analysis conducted by Rent et al. focused exclusively on Hispanic and Black families at a single center in the United States. The authors described lived experiences of explicit miscommunications that deeply impacted patients’ trust of the counseling clinician [69]. Together, these studies illustrate how addressing disparities in communication barriers may improve immediate understanding of fetal CHD and may also help build stronger patient-clinician relationships over time.

### Variation in the Discussion of Non-surgical Options

Another key component of prenatal diagnosis is provision of non-directive, balanced counseling regarding surgical and non-surgical management options of fetal CHD during pregnancy, including termination of pregnancy and perinatal hospice (i.e., hospice birth plan, palliative birth plan, or comfort care without surgical intervention) [70, 71]. However, there remains significant variation in whether non-surgical management options are discussed with parents during fetal cardiologists’ prenatal counseling, which appears to be influenced by clinician and center characteristics. In national survey studies of physicians in North America, 64–80% reported counseling parents about termination [26, 72–75], while 56–72% reported discussing perinatal hospice [26, 72–74, 76]. In analyses focused on those who chose termination or hospice, parents noted that postnatal surgery was an overemphasized option [77]. Thus, clinical consensus is to reduce variation in prenatal counseling regarding management options, which is critically important for families to make fully informed decisions [46, 78]. Variation in counseling on management options may have been further affected by the 2022 *Dobbs v. Jackson Women’s Health Organization* ruling, which revoked the constitutional right to termination and returned authority to regulate termination to individual states, thus impacting access to abortion care in certain states [79, 80]. Early studies have already shown a rise in the live-birth incidence of severe CHD in states with restrictive termination policies [81].

More granular data has demonstrated how clinician bias and variability in knowledge play significant roles in introducing variation in prenatal counseling of management options. Woo et al. conducted a multi-institutional survey study before the *Dobbs* ruling and found that cardiologists and surgeons who would consider termination for themselves were more likely to counsel about termination in practice, compared to those who would not consider termination for themselves [26]. Further, while some clinicians maintain guarded perspectives of the long-term outcomes of single ventricle disease, clinician’s perspectives of outcomes were not associated with counseling practices the way that personal beliefs were [26, 82]. Haxel and Ronai et al. conducted a national survey soon after the *Dobbs* ruling and similarly found that physicians who agree with the statement “some life is better than no life at all” were less likely to value prenatal counseling for both termination and perinatal hospice. Interestingly, there were no significant differences in self-reported counseling rates for termination or perinatal hospice between these pre- and post-*Dobbs* studies [74].

In addition to personal beliefs, familiarity with perinatal hospice may impact whether this option is offered. Balkin et al. conducted a multi-institutional survey evaluating pediatric cardiologists’ attitudes about palliative care (prenatally or post-surgically), and found that 85% agreed that palliative care consultations are valuable, yet only 20–50% of cardiologists felt very competent in discussing life expectancy and goals of care [76]. This suggested that a lack of knowledge or familiarity with the logistics of perinatal hospice are also driving factors for counseling behaviors. While many cardiologists who practice in tertiary or quaternary settings may rely on a palliative care team to deliver this information in a neutral way, a recent benchmarking survey revealed only about 40% of major cardiac centers conduct joint counseling with cardiologists and palliative care specialists [10].

### Future Directions to Reduce Variation in Prenatal Counseling

While the above research highlight some of the root causes for variation in discussions of cardiac anatomy or management options, there remain significant knowledge gaps in whether disparities exist in counseling topics beyond the fetal cardiac anatomy and its management, such as the long-term financial costs of CHD care, or clinician acknowledgement of parental emotions of guilt and helplessness [83]. Perhaps the greatest knowledge gap of all is whether evidence-based interventions can reduce variation in counseling [75]. Until the implementation and dissemination of such evidence-based interventions occurs, variation can still be mitigated by awareness of and attention to (1) parental language barriers, age, and educational attainment, (2) personal beliefs that impact the provision of non-surgical management options, and (3) the logistics of non-surgical management for fetal CHDs, and how to present this information in a neutral, patient-centered manner.

## Conclusion

The key domains for successful prenatal CHD diagnosis include (1) accessing fetal cardiac care, (2) obtaining accurate anatomic assessment, and (3) and receiving comprehensive counseling. Socially defined disparities and/or clinical practice variation affect all three domains. Among neonates undergoing cardiac surgery in the United States, prenatal diagnosis is generally lower among those with public insurance, lower neighborhood socioeconomic status, Hispanic ethnicity, and further distance to care. Outside the United States, high-income countries have higher prenatal diagnosis rates than in low- and middle-income countries, which is likely driven by limited financial resources, workforce differences, and lack of awareness of the utility of fetal echocardiography.

Practice variation by clinician experience, patient factors, and timing of referral can also affect prenatal diagnosis rates. Professional society guidelines have significantly improved this practice variation; these guidelines emphasize the importance of training, skill, multidisciplinary collaboration, and a commitment to quality improvement. There remains substantial variation in the provision of management options during counseling, with clinician bias and knowledge as key drivers.

Regional studies have demonstrated how effective policy and system-level interventions can ameliorate these disparities. Tele-echocardiography and artificial intelligence are exciting frontiers, but more research on the utility and reliability of these technologies is necessary before routine integration. At the clinician level, awareness of and consideration for disparities and practice variation may further facilitate equitable, quality-driven care, contributing to the upward trajectory of prenatal diagnosis rates over time.

## Key References

- Ribeiro ER, Co-Vu J, Quartermain MD, Bonnell LN, Goldberg DJ, Brinkley LM, et al. Variation of Prenatal Detection of Congenital Heart Disease in Infants: Updated Analysis of The Society of Thoracic Surgeons Congenital Heart Surgery Database. Ann Thorac Surg. 2025. doi: 10.1016/j.athoracsur.2025.06.049.
  - ○ Reports recent U.S. rates of prenatal CHD diagnosis in those infants who underwent cardiac surgery. Highlights variation by region.
- Moon-Grady AJ, Donofrio MT, Gelehrter S, Hornberger L, Kreeger J, Lee W, et al. Guidelines and Recommendations for Performance of the Fetal Echocardiogram: An Update from the American Society of Echocardiography. J Am Soc Echocardiogr. 2023;36(7):679-723. doi: 10.1016/j.echo.2023.04.014.
  - ○ Consensus guidelines on indications for, timing of and components of fetal echocardiography.

## Data Availability

No datasets were generated or analysed during the current study.
